# Assessment of a polygenic hazard score for the onset of pre-clinical Alzheimer’s disease

**DOI:** 10.1186/s12864-022-08617-2

**Published:** 2022-05-26

**Authors:** Michael Vacher, Vincent Doré, Tenielle Porter, Lidija Milicic, Victor L. Villemagne, Pierrick Bourgeat, Sam C. Burnham, Timothy Cox, Colin L. Masters, Christopher C. Rowe, Jurgen Fripp, James D. Doecke, Simon M. Laws

**Affiliations:** 1 Australian e-Health Research Centre, CSIRO, Floreat, Western Australia 6014 Australia; 2 Centre for Precision Health, Edith Cowan University, Joondalup, Western Australia 6027 Australia; 3 Collaborative Genomics and Translation Group, School of Medical and Health Sciences, Edith Cowan University, Joondalup, 6027 Western Australia; 4 Australian e-Health Research Centre, CSIRO, Parkville, Victoria 3052 Australia; 5 Department of Molecular Imaging & Therapy and Centre for PET, Austin Health, Heidelberg, Victoria Australia; 6 Curtin Health Innovation Research Institute, Curtin University, Bentley, 6102 Western Australia; 7 Department of Psychiatry, University of Pittsburgh, Pittsburgh, PA USA; 8 Australian e-Health Research Centre, CSIRO, Herston, Queensland 4029 Australia; 9 Florey Institute, The University of Melbourne, Parkville, VIC 3052 Australia

**Keywords:** Alzheimer’s disease, Polygenic hazard score, Brain atrophy, AD onset

## Abstract

**Abstract:**

**Background:**

With a growing number of loci associated with late-onset (sporadic) Alzheimer’s disease (AD), the polygenic contribution to AD is now well established. The development of polygenic risk score approaches have shown promising results for identifying individuals at higher risk of developing AD, thereby facilitating the development of preventative and therapeutic strategies. A polygenic hazard score (PHS) has been proposed to quantify age-specific genetic risk for AD. In this study, we assessed the predictive power and transferability of this PHS in an independent cohort, to support its clinical utility.

**Results:**

Using genotype and imaging data from 780 individuals enrolled in the Australian Imaging, Biomarkers and Lifestyle (AIBL) study, we investigated associations between the PHS and several AD-related traits, including 1) cross-sectional Aβ-amyloid (Aβ) deposition, 2) longitudinal brain atrophy, 3) longitudinal cognitive decline, 4) age of onset. Except in the cognitive domain, we obtained results that were consistent with previously published findings. The PHS was associated with increased Aβ burden, faster regional brain atrophy and an earlier age of onset.

**Conclusion:**

Overall, the results support the predictive power of a PHS, however, with only marginal improvement compared to apolipoprotein E alone.

**Supplementary Information:**

The online version contains supplementary material available at 10.1186/s12864-022-08617-2.

## Introduction

The ε4 allele of the apolipoprotein E (*APOE*) gene is the strongest genetic risk factor for late onset AD [1]. However, polygenic or oligogenic [2] contributions to AD are now widely acknowledged. In recent years, genome wide association studies (GWAS) and next-generation sequencing efforts have facilitated the identification of a number of disease-onset-associated single nucleotide polymorphisms (SNPs) with much smaller effect size [3, 4]. Collectively, these genetic variations could make a significant contribution to age-at-onset AD risk. The combined effect of these inherited genetic variations can be quantified and expressed as a polygenic risk score (PRS). The development and validation of a reliable PRS for AD could contribute to solving a major public health challenge by identifying the age-at-onset of individuals at high risk of developing the disease and therefore enable early screening and preventive therapies [5]. Typically, PRSs are constructed as the weighted sum of allele counts, where the weights correspond to the effects (β coefficients) of each SNP, extracted from a SNPs-disease association analysis (e.g. logistic regression for case-control studies) [6]. The development of PRSs for age-at-onset of AD have demonstrated promising results with prediction capabilities showing over 80% accuracy in some cases [7, 8].

A polygenic hazard score (PHS) approach has also been developed for AD [9]. This PHS goes beyond AD risk prediction by providing estimates of individual age-specific risk for developing AD. Moreover, the PHS has been shown to be associated with Aβ-amyloid (Aβ) accumulation, accelerated cognitive decline and neurodegeneration in susceptible brain regions. In this study we aim to assess the useability and replicability of PHS in an independent cohort.

## Materials and methods

### Sample population

The study used data from the Australian Imaging, Biomarkers and Lifestyle (AIBL) cohort, of which the design, enrolment process, neuropsychological assessments and diagnostic criteria have been previously described [10]. Of the 1572 participants enrolled in the AIBL study, we restricted the analysis to individuals having both genotype and imaging data available (*N* = 780). Longitudinal data was collected every 18 months over multiple years (mean = 4.8 years, SD = 2.1). Participants were classified as those with Mild Cognitive Impairment (MCI) [11] or AD [12] when the clinical criteria for diagnosis were met. In the absence of these features a classification of Cognitively Normal (CN) was given by a clinical review panel, blinded to Aβ-PET status (see below). Ethics approval for the AIBL study and all experimental protocols was provided by Austin Health, St Vincent’s Health, Hollywood Private Hospital and Edith Cowan University. All experiments and methods were carried out in accordance with approved guidelines and regulations and all volunteers gave written informed consent before participating in the study.

### MRI and PET imaging

All subjects underwent a 3 T MRI and Aβ-PET imaging. T1 MPRAGE MRI was obtained at 3 T using the Alzheimer’s Disease Neuroimaging Initiative (ADNI) magnetization-prepared rapid gradient echo (MPRAGE) protocol, with in-plan resolution of 1 × 1 mm and 1.2 mm slice thickness. Freesurfer was used to estimate all cortical volumes from the T1 [13]. All volumes were corrected for age and ICV using a regression approach and reference population composed of healthy subjects (CN, Aβ negative, MMSE> 28, CDR = 0, *APOE* non-ε4). Left and right volumes were averaged.

Aβ-PET imaging was performed with one of five radiotracers: [^11^C]-PiB, [^18^F]-flutemetamol (FLUTE), [^18^F]-florbetapir (FBP), [^18^F]-florbetaben (FBB), and [^18^F]-NAV4694 (NAV). A 20- minute acquisition was performed 50 minutes post-injection of PiB, NAV and FBP, and 90 minutes post-injection of FLUTE and FBB. Due to the difference in SUVR dynamic ranges of each Aβ tracer, the Centiloid (CL) scale was used to provide a standard scale for Aβ-PΕΤ quantification [14], with 0 representing the typical Aβ-PΕΤ in young controls, and 100 the typical Aβ-PET in mild AD patients. Values equal to or above 20 CL were considered representative of abnormal levels of Aβ deposition [15]. CL were generated using CapAIBL software [14] and estimated in three regions of interest (whole neocortex, frontal and posterior cingulate). CL values were also projected onto the individual cortical surface and then transferred to a cortical atlas where statistical analyses were performed [16].

### Cognitive scores

All AIBL participants complete a battery of neuropsychological tests as previously described [10]. The resulting data were used to calculate cognitive composite scores to assess recognition memory, executive function and episodic recall memory. Briefly, the composites were computed by standardising the outcome measure for each neuropsychological test to be included, using the baseline mean and standard deviation for the cognitively normal sample, then averaging those standardised scores. Each composite consists of the following tests; recognition memory (California Verbal Learning Test Second Edition and Rey Complex Figure Test), executive function (Controlled Oral Word Association Test and Category Switching), and episodic recall memory (California Verbal Learning Test Second Edition, Logical Memory II, and Rey Complex Figure Test) (Harrington reference). Clinical Dementia Rating (CDR) sum of boxes (CDRSB) score was also used to assess clinical progression.

### Age of onset definition

The age of onset of abnormal levels of amyloid deposition was determined as follows: First a progression curve giving for Aβ-amyloid deposition as a function of disease progression time was constructed as described in [17, 18]. Then, the participants’ age of onset was estimated by using the progression curve to calculate the elapsed time between a participant passing the CL threshold and reaching their mean longitudinal Aβ-amyloid levels. Finally, this value was subtracted from their mean longitudinal age to get the age at onset.

### Genetic data and Polygenic hazard score

Genome wide genetic data was ascertained from the OmniExpressHumanExome+ BeadChip (Illumina, USA) as previously described [10]. A polygenic hazard score was then derived from this genetic data for each individual following the methodology described previously [9]. Briefly, the approach consists in three steps. First, a list of 1854 SNPs implicated with AD (*p*-values < 10^− 5^) was extracted from the published summary statistics (*p*-values and odds ratios) generated by the IGAP consortium [4]. Second, a forward stepwise regression to identify a subset of 31 SNPs, in addition to the two *APOE* variants, that were associated with AD age of onset. Finally, for each patient, a polygenic hazard score predicting the individual’s risk of developing AD, given their polygenic profile and age was derived. In the analyses we assessed the PHS as a continuous measure, quantifying individual risk for AD, and as a dichotomous variable (high and low). To directly replicate the findings from Tan et al. [19], we used the same grouping method, defining high PHS by 1 standard deviation (SD) above the mean and low PHS by 1 SD below the mean (Supplementary Fig. 1).

### Statistical analyses

#### Association with cross-sectional Aβ deposition

We used linear regression to investigate the relationship between PHS and regional brain Aβ deposition at baseline. Three main areas were investigated: neocortical, frontal cortex and posterior cingulate. In this cross-sectional analysis, we controlled for age, gender, level of education in years and *APOE* ε4 status (0 = no ε4 allele, 1 = 1 or 2 ε4 alleles). To evaluate the contribution of PHS and *APOE* ε4 status in the linear regression, we used likelihood ratio tests to compare models with and without these terms.

To further assess the association between PHS and Aβ deposition, beyond the role *APOE*, we performed the same analysis in two sub-cohorts containing exclusively *APOE* ε4 carriers (*N* = 278; CN = 161, MCI = 58, AD = 59) and non-carriers (*N* = 502; HC = 412, MCI = 66, AD = 24). In these subsequent analyses, we used the same linear regression, however, we did not control for *APOE* ε4 status due to the lack of variation in these sub-populations. All the results were adjusted for multiple comparisons using false discovery rate (FDR). The same analysis was performed at a vertex level on a template cortical surface.

#### Association with regional brain atrophy

We used linear mixed-effects models to evaluate the relationship of PHS with longitudinal volume change in 33 regions of interest from the Desikan-Killiany atlas in Freesurfer [20]. As volumes were previously controlled for intra-cranial volume (ICV) and age using a healthy sub-population we only controlled for gender, level of education in years and *APOE* ε4 status (0 = no ε4 allele, 1 = 1 or 2 ε4 alleles). In these analyses, we also controlled for sex, education, and *APOE* status. We then examined the simple effects by comparing slopes of volume loss over time for individuals at high (+ 1 SD) and low (− 1 SD) levels of PHS [19, 21].

#### Association with longitudinal cognitive decline

For comparative purposes, we used the same linear mixed effects models as described in the original Desikan paper [19]. The only deviation made from this model was the use of the CL value from the frontal cortex instead of the standard uptake volume ratio (SUVR). Therefore, the final linear mixed effect model was defined as follows:

$${\Delta}_c={\beta}_0+{\beta}_1\ PHS\ast Time+{\beta}_2\ Centiloid(fc)\ast Time+{\beta}_3\ enthorinal\ cortex\ volume\ast Time+{\beta}_4\ Baseline\ Age\ast Time+{\beta}_5\ Sex\ast Time+{\beta}_6\ Education\ast Time+{\beta}_7\ APOE\ast Time+\left(1| Patient\right)$$

This model was used to investigate the association between PHS and cognitive decline and clinical progression rate (represented as Δ_*c*_) across four measures: *recognition*, *executive function*, *episodic recall* and *CDR-SB*. In this model, *Time* represented the number of years since the baseline visit. The *APOE* term indicated the presence/absence of *APOE* ε4 allele, encoded as a binary variable (0 = no ε4 allele, 1 = 1 or 2 ε4 alleles) and the term (1| *Patient*) corresponded to the random intercept. Continuous variables were centred and scaled in all the analyses. Further, to assess the original model and limit potential over-specification, we used a stepwise variable selection approach (backward selection) and identified a reduced model based on superior model fit (Akaike information criterion).

#### Association with age of onset of abnormal levels of Aβ deposition

Cox proportional hazards models of survival were performed to compare the time taken to reach abnormal levels of neocortical Aβ between participants with low versus high PHS scores (threshold at 1.04), adjusted baseline age, gender and years of education. The definition for survival time was the number of years between birth and a) having a PET scan indicating abnormal levels of Aβ (classed here an event, age of onset), b) withdrawing from the study (censored), or c) the last completed follow-up examination without an event (censored). For some individuals it was necessary to impute the date at which their Aβ levels became abnormal as previously published [22]. The age at which 50% of the cohort (median age) reached abnormal levels of Aβ, was reported.

## Results

### Demographics

The study was conducted on a cohort of 780 participants enrolled in the AIBL study [23] (422 females, 358 males). Population demographic information is displayed in Table 1. Comparing demographic and clinical characteristics between diagnoses, AD participants were older than CN and MCI participants (AD: 75.0 [SD: 7.85], MCI: 75.6 [SD: 7.13], CN: 72.9 [SD: 6.12], *p* < 0.001) and were more likely to carry at least one copy of *APOE* ε4 allele (AD: 71.1%, MCI: 46.8%, HC: 28.1%, p < 0.001). As expected, the PHS were significantly higher in AD participants (AD: 0.847 [SD: 0.941], MCI: 0.415 [SD: 0.967], CN: 0.0271 [SD: 0.763], *p* = 8.25e^− 08^). This result was consistent in the sub-population consisting of *APOE* ε4 carriers only (AD: 1.33 [SD: 0.62], MCI: 1.29 [SD: 0.66], CN: 1.04 [SD: 0.55], *p* = 9.55e^− 4^). However, in the *APOE* ε4 non-carrier subgroup, the difference of PHS across clinical classification was not seen (AD: -0.35 [SD: 0.31], MCI: -0.35 [SD: 0.34], CN: -0.37 [SD: 0.36], *p* = 0.895).

**Table 1:** Population characteristics

	CN (***N*** = 573)	MCI (***N*** = 124)	AD (***N*** = 83)	***P***-value
**Sex**				
Female	318 (55.5%)	56.0 (45.2%)	48.0 (57.8%)	0.0859
Male	255 (44.5%)	68.0 (54.8%)	35.0 (42.2%)	
**Age (years)**				
Mean (SD)	72.9 (6.12)	75.6 (7.13)	75.0 (7.85)	< 0.001
Median [Min, Max]	72.7 [60.0, 93.6]	76.3 [56.1, 95.4]	74.8 [57.8, 93.2]	
***APOE*ε4**				
Absent	412 (71.9%)	66.0 (53.2%)	24.0 (28.9%)	< 0.001
Present	161 (28.1%)	58.0 (46.8%)	59.0 (71.1%)	
**Education (years)**				
0–6	1.00 (0.2%)	1.00 (0.8%)	2.00 (2.4%)	0.0148
7–12	41.0 (7.2%)	17.0 (13.7%)	11.0 (13.3%)	
9–12	213 (37.2%)	50.0 (40.3%)	29.0 (34.9%)	
13–15	118 (20.6%)	22.0 (17.7%)	20.0 (24.1%)	
15+	200 (34.9%)	34.0 (27.4%)	21.0 (25.3%)	
**PHS**				
Mean (SD)	0.0271 (0.763)	0.415 (0.967)	0.847 (0.941)	< 0.001
Median [Min, Max]	−0.178 [−1.60, 2.59]	0.162 [−1.26, 3.12]	1.01 [−1.06, 2.91]	

*P* values determined by Fisher’s test (*APOE* ε4 and Gender), t-test (age), and Chi square analyses

*N* number, *CN* cognitively normal, *MCI* mild cognitive impairment, *AD* Alzheimer’s disease, *APOE* ε4 apolipoprotein ε4 allele

### Association with cross-sectional Aβ deposition

Using linear regression to evaluate the relationship between PHS and Aβ burden at baseline, we found that increased PHS values were associated with higher Aβ deposition. Similar results were obtained when looking at CL values specifically in the three areas investigated, neocortical, frontal cortex and posterior cingulate, with stronger effects systematically obtained in the posterior cingulate region, followed by the frontal cortex and then the neocortical region (Table 2). These results were consistent in the sub-populations composed exclusively of *APOE* ε4 carriers and non-carriers, although with less significant effects. We then used likelihood ratio tests to determine if the addition of PHS and *APOE* ε4 status in a model resulted in improvements in the fit. The addition of *APOE* ε4 status (β = 39.5, SE = 2.8, *p* = 8.05e^− 39^) and PHS (β = 24.2, SE = 1.55, *p* = 3.74e^− 48^), individually, both resulted in statistically significant improvements from a base model controlling for age, gender and education only. However, the addition of PHS in a model already controlling for *APOE* ε4 status was not significant in any of the three areas investigated (frontal cortex, *p* = 0.066; posterior cingulate, *p* = 0.1306; neocortical, *p* = 0.08).

**Table 2:** Association between PHS and cross-sectional Aβ deposition

Population	N [CN/MCI/AD]	Region	beta	SE	CI 95%	FDR-adjusted p
Whole Cohort	780 [573/124/83]	neocortical	19.98	2.91	[14.3–25.7]	1.44E-10
		posterior cingulate	25.54	3.61	[18.4–32.6]	7.41E-11
		frontal cortex	21.19	3.15	[15.0–27.4]	2.51E-10
*APOE* ε4 carriers	278 [161/58/59]	neocortical	25.28	4.00	[17.4–33.2]	5.17E-08
		posterior cingulate	33.06	4.94	[23.3–42.8]	7.88E-09
		frontal cortex	26.63	4.38	[18.0–35.3]	1.31E-07
*APOE* ε4 non carriers	502 [412/66/24]	neocortical	12.61	4.43	[3.90–21.3]	1.25E-02
		posterior cingulate	14.28	5.53	[3.42–25.1]	2.43E-02
		frontal cortex	13.62	4.77	[4.24–23.0]	1.25E-02

### Association with regional brain atrophy

**Fig. 1:**
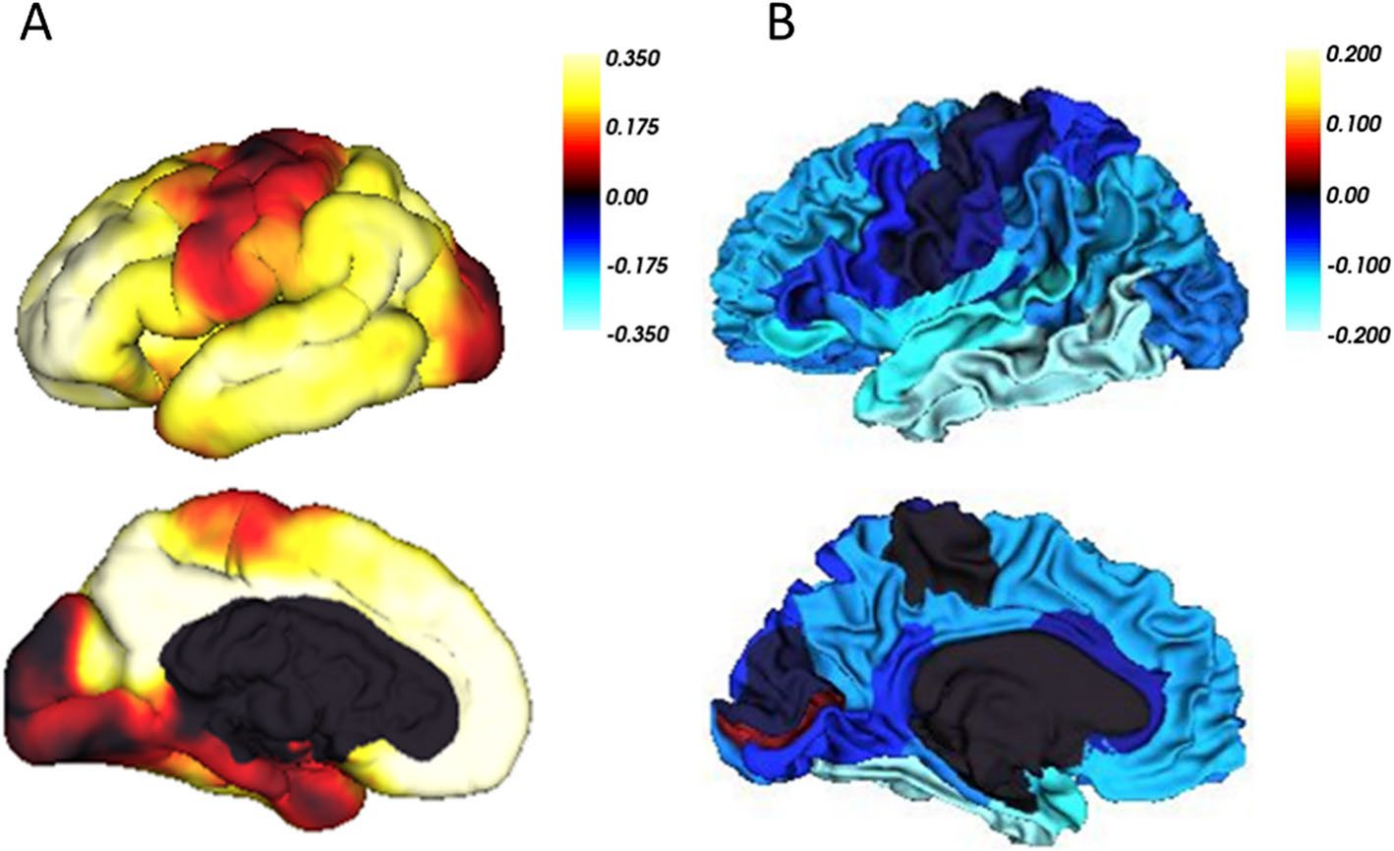
PHS is associated with local Aβ and cortical atrophy. Beta estimates of (**A**) the associations of PHS with cross-sectional voxel-wise CL and (**B**) Longitudinal change in regional cortical volumes in individuals with high (1 SD above mean, ∼ 84 percentile) PHS

In the linear-mixed effect analysis, PHS was significantly associated with cortical volume changes in most regions of interest. The effects were strongest in regions from the AD cortical signature, including temporal lobe, entorhinal cortex, posterior cingulate and precuneus. The cortical atrophy was fastest in individuals with high PHS (Fig. 1). However, when looking at non-ε4 carriers only, none of the associations between PHS and cortical atrophy remained significant after controlling for multiple comparisons. In addition, the effect sizes were considerably reduced (Supplementary Fig. 2).

### Association with cognitive decline

In the linear mixed model analyses, we did not find any significant association between PHS and cognitive decline. The analyses were restricted to non-AD individuals (CN + MCI and CN only) and the results were consistent across the three cognitive domains investigated (Table 3). Further, comparing individuals with high PHS against low PHS, there was no significant difference in rate of cognitive decline between the two groups. Likelihood ratio tests comparing linear-mixed effects models with and without the PHS term, showed that the presence of PHS in the model resulted in minor improvement in CDR-SB (*X*^*2*^ = 6.29, *p* = 0.043) and Episodic recall (*X*^*2*^ = 6.58, *p* = 0.037). However, the addition of PHS did not result in a better fit in the model predicting Executive function (*X*^*2*^ = 2.83, *p* = 0.24).

**Table 3:** Associations between PHS and cognitive decline

Population	N	Domain	beta	SE	CI 95%	p
CN + MCI	697	CDR SoB	0.004	0.014	[0.023–0.03]	0.747
		Episodic Recall	0.007	0.011	[−0.014–0.028]	0.519
		Executive Function	− 0.023	0.014	[− 0.05–0.003]	0.086
CN	573	CDR SoB	0.023	0.017	[−0.01–0.056]	0.174
		Episodic Recall	0.001	0.013	[−0.024–0.026]	0.948
		Executive Function	−0.018	0.015	[−0.047–0.011]	0.225

### Association with age of onset of abnormal levels of *Aβ* deposition

**Fig. 2:**
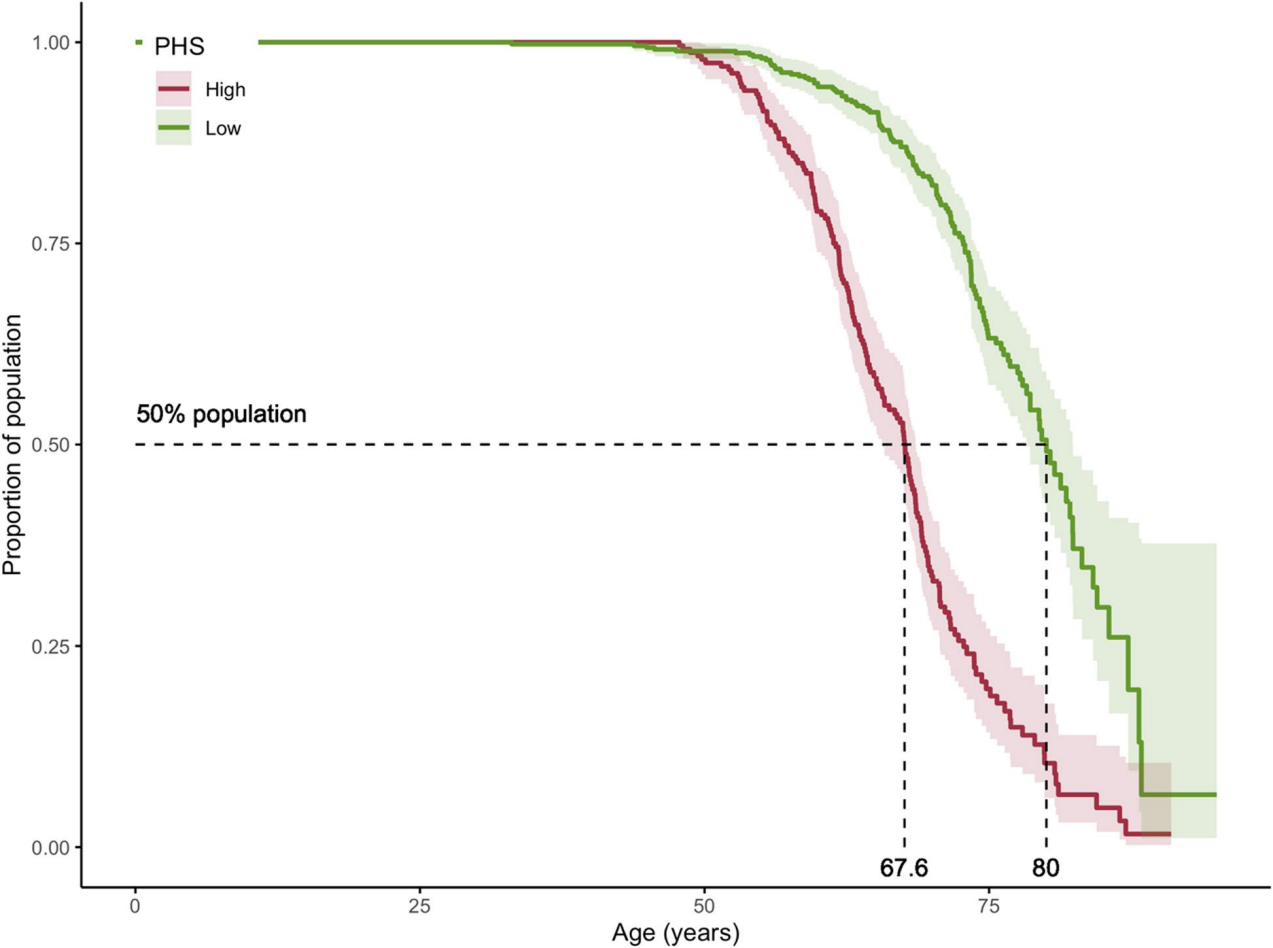
Kaplan-Meier plot showing the age of onset, defined as the age at which individual reach an abnormal level of Aβ (CL ≥ 20). The population was stratified by PHS, high (mean + 1 SD) versus low (mean – 1 SD). Shaded areas indicate 95% confidence intervals

The age of onset of individuals with a high PHS (> 1.04) was significantly lower than individuals with a low PHS (<− 0.67). The median age at which AIBL participants with a high PHS reached abnormal levels of Aβ (Fig. 2) was 67.6 years (CI 95% [65.6, 68.6]), 12.4 years earlier than those with a low PHS (80 years, CI 95% [78.3, 82.4]). The hazard ratio comparing the high PHS versus the low PHS group was 3.9 (CI 95% [3.1, 4.9], log rank test *p* = 3.72e^− 17^). Restricting this analysis to ε3/ε3 individuals only, the high PHS group still had an earlier age of onset (73.9 years, CI 95% [72.4, 78.2]) compared to the low PHS population (82.4 years, CI 95% [76.8.4, 84.2]), with a hazard ratio of 1.8 (CI 95% [0.9, 3.2], log rank test *p* = 0.072).

## Discussion

Over the last decade, GWAS have clearly demonstrated that common complex diseases are typically associated with hundreds or thousands of genetic markers, collectively contributing to disease risk. This highly polygenic underpinning has been utilised in PRS by aggregating the effect of multiple genetic variants into a single score to predict disease risk. PRS have shown predictive values in several complex disorders, however, the approach does not account for age of onset, which is critical for neurodegenerative disease such as AD. The recent development of polygenic hazard score overcomes this limitation by predicting individuals’ age-specific risk of AD development [9]. In this study, we attempted to replicate the findings from Tan et al. [19] and assess the utility of a PHS in an independent cohort. While investigating the relationship between PHS and cross-sectional Aβ deposition, we found that the PHS had slightly better predicting capabilities than using *APOE* ε4 status alone. This result was expected considering the effects of the *APOE* ε2 and ε4 alleles were already accounted for in the calculation of the PHS. However, the marginal improvement in models to predict Aβ deposition observed when using PHS, suggests that the number of *APOE* ε4 alone can provide comparable predictive information. The major contribution of *APOE* in the PHS definition is evident when looking at the stratified score distribution (Supplementary Fig. 1). Individuals falling in the *low* PHS bracket (1 SD below the mean) are all ε4 non-carriers, while all ε4/ε4 individuals, conversely, are found in the *high* PHS group (1 SD above the mean). Nonetheless, we were able to find associations between PHS and regional Aβ deposition and brain atrophy in non-ε4 individuals. Although with relatively small effect sizes, these associations are consistent with the findings reported by Tan et al. [19] and support the claim that the PHS can provide predicting capabilities beyond *APOE* status.

Investigating cognitive domains, we did not find any evidence of association between PHS and longitudinal cognitive decline. These analyses were performed on non-demented individuals (*N* = 697) with a proportion of 82% CN and 18% MCI. This ratio was very different from the study cohort utilised in Tan et al., which consisted of about 36% CN and 64% MCI. Considering that healthy individuals have a much less pronounced cognitive decline trajectory, the over-representation of CN in our dataset could have impacted the results. Further, the linear mixed model used in this study was slightly different than the one presented in the original paper. Specifically, we used the CL values instead of the frontal florbetapir SUVR. Although, it is not expected that this deviation would generate differing results, they could have impacted and decreased the strength of the associations between PHS and cognitive decline.

Investigating the associations between PHS and age of onset, we found that individuals with a *high* PHS had a significantly younger age of onset (67.6 years) than *low* PHS individuals (80 years). Among the *APOE* ε3/ε3 population, *high* PHS individuals had an expected age of onset approximatively 8 years younger than *low* PHS individuals. Although the difference was less noticeable in the ε3/ε3 population, this result showed that the polygenic information, beyond the *APOE* ε4 allele, was useful for predicting the age of disease onset.

Compared to traditional polygenic risk scores, which provide a lifetime risk of developing a disease, the polygenic hazard scores provide prediction on age-specific risk of disease development. This estimation of instantaneous risk for developing AD is valuable additional information, as it could improve monitoring disease progression and facilitate timely intervention. In this study, we showed that the PHS had utility in predicting abnormal Aβ deposition, brain atrophy and age of disease onset. However, the results suggested that *APOE* genotype alone could provide comparable predictive capabilities, suggesting that *APOE* remains the main component of the PHS. This disproportionate contribution is mainly due to the large weight attributed to *APOE* alleles, which shadows the less extensive effects of the other disease-associated variants utilised in the score calculation. This is the case in both PRS and PHS approaches and reflect the difficulty in classifying AD genetic risk above and beyond *APOE* ε4. Recent studies have suggested that the use of less stringent significance threshold for SNPs selection could results in better performing scores [7, 8, 24]. This strategy could potentially further improve the prediction accuracy of the PHS. However, caution must be taken as the inclusion of many SNPs could lead to over-specification and result in a score that performs poorly in other cohorts. Furthermore, in addition to non-modifiable factors, PHS and PRS could both be enriched with the introduction of lifestyle and environmental factors, known to be associated with the disease. Moving forward, we can anticipate that combining genetic, lifestyle and environmental components will facilitate the development of more refined and personalised risk profiles that will become relevant in clinical settings.

## Supplementary Information

**Additional file 1: Supplementary Figure 1**. A, Distribution of PHS in the whole cohort. High and low PHS were defined as 1 SD above the mean and 1 SD below the mean respectively. B, Distribution of PHS stratified by number of APOE e4 alleles (0 = e2/e3, e2/e2, e3/e3; 1 = e4/e3, e4/e2 ; 2 = e4/e4).

**Additional file 2: Supplementary Figure** 2. PHS is associated with local Ab and cortical atrophy in non-ε4 carrier. Beta estimates of (A) the associations of PHS with cross-sectional voxel-wise Centiloid and (B) Longitudinal change in regional cortical volumes in non-ε4 carrier individuals.

**Additional file 3: Supplementary Table 1**. Beta estimates of the interaction between PHS and time on longitudinal regional cortical volume change controlling for *APOE* status.

## Data Availability

All data and samples used in this study are derived from the Australian Imaging, Biomarkers and Lifestyle (AIBL). All AIBL data, and that specific to this study, are publicly accessible to all interested parties through an Expression of Interest procedure and is governed by the AIBL Data Use Agreement. For more information, please visit https://aibl.csiro.au/awd/.
