# Supplementary figures and images for: Assessment of a polygenic hazard score for the onset of pre-clinical Alzheimer’s disease
Source: BMC Genomics. 2022 May 26;23:401. doi: 10.1186/s12864-022-08617-2 (PMC9134703; doi:10.1186/s12864-022-08617-2)

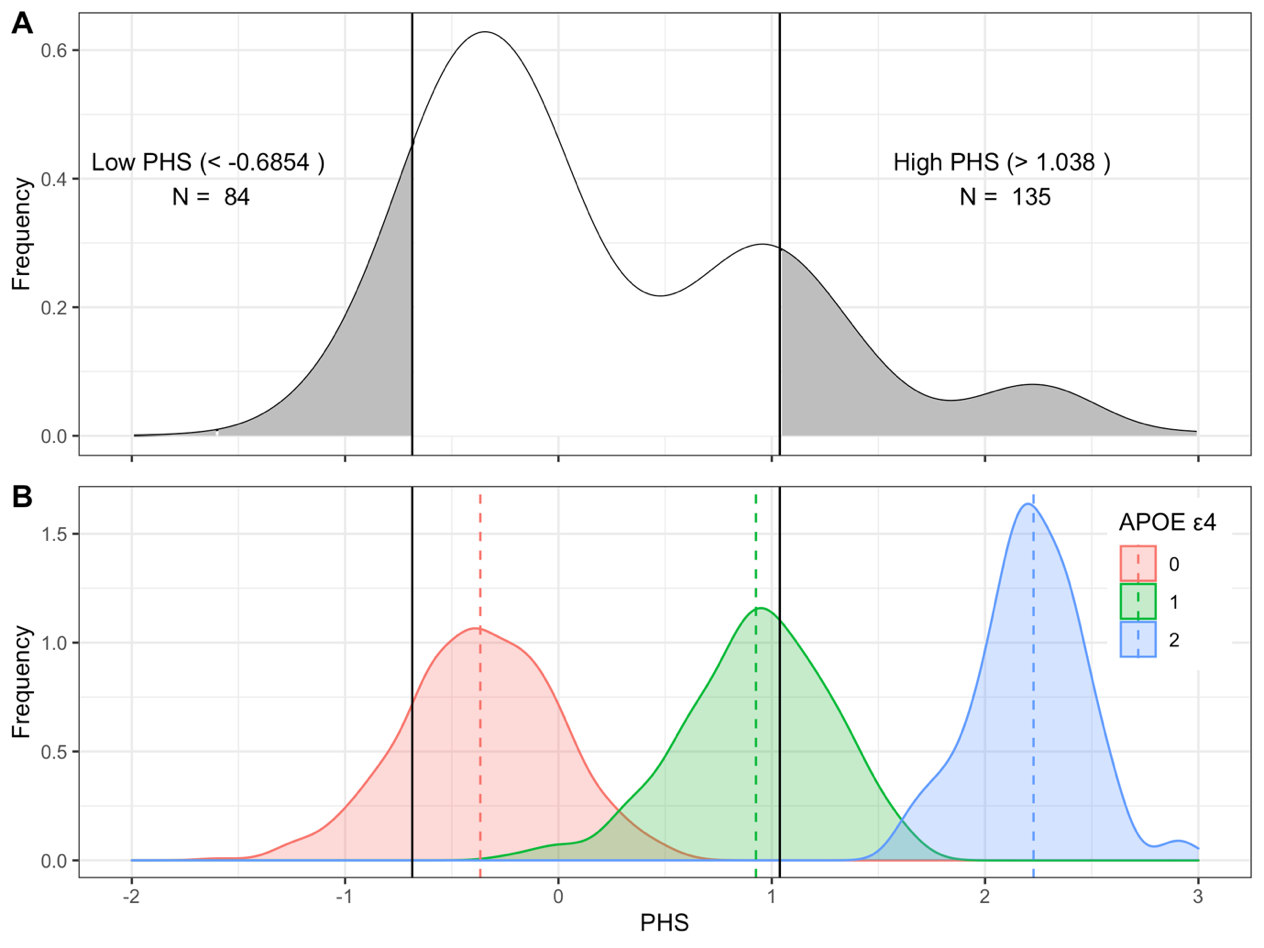

Supplement: Supplementary file 1 — Additional file 1: Supplementary Figure 1. A, Distribution of PHS in the whole cohort. High and low PHS were defined as 1 SD above the mean and 1 SD below the mean respectively. B, Distribution of PHS stratified by number of APOE e4 alleles (0 = e2/e3, e2/e2, e3/e3; 1 = e4/e3, e4/e2 ; 2 = e4/e4). [file 12864_2022_8617_MOESM1_ESM.docx]

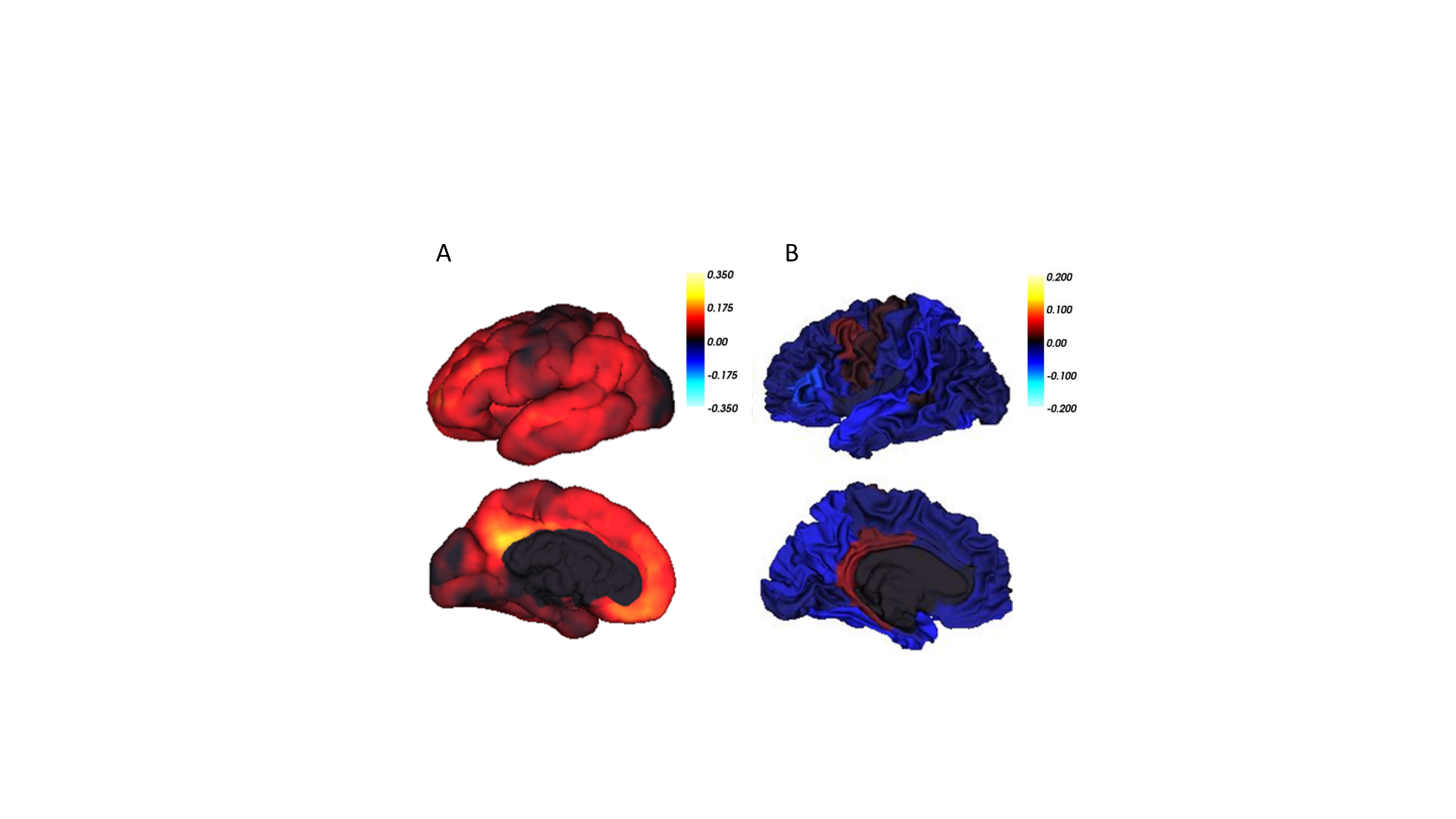

Supplement: Supplementary file 2 — Additional file 2: Supplementary Figure 2. PHS is associated with local Ab and cortical atrophy in non-ε4 carrier. Beta estimates of (A) the associations of PHS with cross-sectional voxel-wise Centiloid and (B) Longitudinal change in regional cortical volumes in non-ε4 carrier individuals. [file 12864_2022_8617_MOESM2_ESM.docx]
