# Supplementary material for: Assessment of a polygenic hazard score for the onset of pre-clinical Alzheimer’s disease
Source: BMC Genomics. 2022 May 26;23:401. doi: 10.1186/s12864-022-08617-2 (PMC9134703; doi:10.1186/s12864-022-08617-2)
Supplement: Supplementary file 3 — Additional file 3: Supplementary Table 1. Beta estimates of the interaction between PHS and time on longitudinal regional cortical volume change controlling for APOE status. [file 12864_2022_8617_MOESM3_ESM.docx]

| **Region of Interest (ROI)** | **PHS*time Beta** | **95% CI** | **p values** | **FDR-adjusted p** |
| --- | --- | --- | --- | --- |
| Middletemporal | -0.19 | (-0.24--0.13) | 4.27E-10 | 4.6970E-09 |
| Inferiortemporal | -0.18 | (-0.23--0.13) | 1.44E-11 | 4.7520E-10 |
| Fusiform | -0.18 | (-0.24--0.13) | 4.42E-11 | 7.2930E-10 |
| Entorhinal | -0.16 | (-0.21--0.11) | 4.28E-10 | 3.5310E-09 |
| Superiortemporal | -0.14 | (-0.19--0.09) | 7.45E-08 | 4.9170E-07 |
| Temporalpole | -0.14 | (-0.20--0.08) | 1.43E-05 | 4.7190E-05 |
| Parsorbitalis | -0.13 | (-0.19--0.08) | 6.15E-06 | 2.5369E-05 |
| Inferiorparietal | -0.11 | (-0.16--0.07) | 1.41E-06 | 7.7550E-06 |
| Posteriorcingulate | -0.11 | (-0.15--0.06) | 4.14E-06 | 1.9517E-05 |
| Precuneus | -0.11 | (-0.16--0.05) | 0.00015133 | 0.0005 |
| Rostralmiddlefrontal | -0.11 | (-0.18--0.05) | 0.00053166 | 0.0013 |
| Superiorfrontal | -0.11 | (-0.18--0.04) | 0.00359447 | 0.0056 |
| Medialorbitofrontal | -0.11 | (-0.19--0.03) | 0.00544733 | 0.0078 |
| Bankssts | -0.1 | (-0.14--0.05) | 8.68E-06 | 3.1827E-05 |
| Supramarginal | -0.1 | (-0.16--0.05) | 0.00017424 | 0.0005 |
| Lateraloccipital | -0.1 | (-0.15--0.04) | 0.00171894 | 0.0033 |
| Lateralorbitofrontal | -0.1 | (-0.16--0.03) | 0.00271302 | 0.0050 |
| Parahippocampal | -0.1 | (-0.16--0.03) | 0.00325593 | 0.0054 |
| Frontalpole | -0.09 | (-0.16--0.02) | 0.01208879 | 0.0160 |
| Transversetemporal | -0.08 | (-0.12--0.03) | 0.00157147 | 0.0032 |
| Parstriangularis | -0.08 | (-0.13--0.03) | 0.00316401 | 0.0055 |
| Isthmuscingulate | -0.07 | (-0.11--0.03) | 0.00070644 | 0.0017 |
| Lingual | -0.07 | (-0.11--0.03) | 0.00138273 | 0.0030 |
| Parsopercularis | -0.07 | (-0.12--0.02) | 0.00452006 | 0.0068 |
| Caudalmiddlefrontal | -0.07 | (-0.12--0.02) | 0.008298 | 0.0114 |
| Superiorparietal | -0.07 | (-0.13--0.01) | 0.01885683 | 0.0239 |
| Rostralanteriorcingulate | -0.04 | (-0.07-0.00) | 0.05359525 | 0.0632 |
| Caudalanteriorcingulate | -0.03 | (-0.06-0.00) | 0.04120719 | 0.0504 |
| Postcentral | -0.03 | (-0.10-0.04) | 0.37337076 | 0.4107 |
| Cuneus | -0.01 | (-0.05-0.03) | 0.6682939 | 0.7114 |
| Precentral | -0.01 | (-0.08-0.06) | 0.7794068 | 0.8038 |
| Paracentral | 0 | (-0.06-0.06) | 0.96900947 | 0.9690 |
| Pericalcarine | 0.04 | (0.00-0.08) | 0.08033686 | 0.0914 |
